# Healthcare at the Crossroads: The Need to Shape an Organizational Culture of Humanistic Teaching and Practice

**DOI:** 10.1007/s11606-018-4470-2

**Published:** 2018-05-08

**Authors:** Elizabeth A. Rider, MaryAnn C. Gilligan, Lars G. Osterberg, Debra K. Litzelman, Margaret Plews-Ogan, Amy B. Weil, Dana W. Dunne, Janet P. Hafler, Natalie B. May, Arthur R. Derse, Richard M. Frankel, William T. Branch

**Affiliations:** 1 Department of Pediatrics, Harvard Medical School, Boston, MA USA; 2 Institute for Professionalism & Ethical Practice, and Division of General Pediatrics, Department of Medicine, Boston Children’s Hospital, Boston, MA USA; 3 Division of General Internal Medicine, Department of Medicine, Medical College of Wisconsin, Milwaukee, WI USA; 4 Department of Medicine (Teaching), Stanford University School of Medicine, Palo Alto, CA USA; 5 Department of Medicine, Indiana University School of Medicine, Indianapolis, IN USA; 6 Division of General, Geriatric, Palliative and Hospital Medicine, Department of Medicine, University of Virginia School of Medicine, Charlottesville, VA USA; 7 Department of Internal Medicine, University of North Carolina School of Medicine, Chapel Hill, NC USA; 8 Department of Internal Medicine, Yale University School of Medicine, New Haven, CT USA; 9 Department of Pediatrics, Yale University School of Medicine, New Haven, CT USA; 10 Center for Bioethics and Medical Humanities, Institute for Health and Equity, Medical College of Wisconsin, Milwaukee, WI USA; 11 Department of Emergency Medicine, Medical College of Wisconsin, Milwaukee, WI USA; 12 Education Institute, Cleveland Clinic, Cleveland, OH USA; 13 Division of General Medicine and Geriatrics, Department of Medicine, Emory University School of Medicine, Atlanta, GA USA

**Keywords:** humanism, organizational culture, faculty development, burnout, leadership, values, compassionate healthcare

## Abstract

**Background:**

Changes in the organization of medical practice have impeded humanistic practice and resulted in widespread physician burnout and dissatisfaction.

**Objective:**

To identify organizational factors that promote or inhibit humanistic practice of medicine by faculty physicians.

**Design:**

From January 1, 2015, through December 31, 2016, faculty from eight US medical schools were asked to write reflectively on two open-ended questions regarding institutional-level motivators and impediments to humanistic practice and teaching within their organizations.

**Participants:**

Sixty eight of the 92 (74%) study participants who received the survey provided written responses. All subjects who were sent the survey had participated in a year-long small-group faculty development program to enhance humanistic practice and teaching. As humanistic leaders, subjects should have insights into motivating and inhibiting factors.

**Approach:**

Participants’ responses were analyzed using the constant comparative method.

**Key Results:**

Motivators included an organizational culture that enhances humanism, which we judged to be the overarching theme. Related themes included leadership supportive of humanistic practice, responsibility to role model humanism, organized activities that promote humanism, and practice structures that facilitate humanism. Impediments included top down organizational culture that inhibits humanism, along with related themes of non-supportive leadership, time and bureaucratic pressures, and non-facilitative practice structures.

**Conclusions:**

While healthcare has evolved rapidly, efforts to counteract the negative effects of changes in organizational and practice environments have largely focused on cultivating humanistic attributes in individuals. Our findings suggest that change at the organizational level is at least equally important. Physicians in our study described the characteristics of an organizational culture that supports and embraces humanism. We offer suggestions for organizational change that keep humanistic and compassionate patient care as its central focus.

**Electronic supplementary material:**

The online version of this article (10.1007/s11606-018-4470-2) contains supplementary material, which is available to authorized users.

## INTRODUCTION

One of medicine’s great traditions is humanistic care of those who are suffering.^1^^–^6 Deep-seated personal commitment to incorporate human values like caring, compassion, and respect into every health care relationship defines medical humanism and is imbedded within the fabric of medical professionalism.^2^^–^4^,^^7^^–^15 Yet, while remarkable technological advances have influenced what we can do *to* patients, much of what patients want and expect from their doctors remains within the humanistic realm of *being with* patients when they are suffering. This includes listening, helping with difficult decisions, and navigating their illness trajectories.^1^^–^15

Healthcare has evolved rapidly, resulting in significant changes in organizational and practice environments. Economic forces and commercial interests now drive the healthcare industry to focus on clinical productivity, efficiency, performance metrics and regulations, pushing physicians to see higher volumes of patients with less time for each.^16^^,^^17^ Time spent in meaningful interactions with patients has diminished, compromising the traditional patient–doctor relationship.

Over half of US physicians now experience professional burnout.^18^^,^^19^ Healthcare professionals’ stress and burnout inhibit forming therapeutic relationships and detract from the patient experience and quality of care.^20^ Incongruence between personal and health system values and work overload contribute significantly to physician burnout, whereas value congruence significantly predicts professional efficacy in addition to well-being of physicians.^18^^,^^21^

Medical educators’ efforts to counter the decline in humanistic care have largely focused on cultivating humanistic attributes in individual physicians.^22^^,^^23^ Such efforts have included curricular and programmatic interventions, such as courses teaching ethics and professionalism, mindfulness, and well-being and group support.^24^ At the same time, it is clear that organizational factors play a central role in physician stress and the development of burnout.^21^^,^^24^ Organizational factors influence how individuals act, their responses to new situations, what they pay attention to, and significantly impact patient safety and quality of care.^25^^,^^26^

For these reasons, we studied factors at the organizational level that may be key to either promoting or impeding the compassionate, humanistic care that most physicians strive to deliver. In this qualitative study, we asked physicians at eight medical schools (Box 1), who had participated in a one-year small-group faculty development program in humanism,^27^^–^31 to identify organizational-level factors that either promoted or impeded their ability to practice humanistically. We chose to study physicians who were promising and respected teachers with an interest in humanism, whom we expected would have insights into achieving humanistic practice.

Box 1 Institutions Participating in Program and Survey

**Table:** 

• Harvard Medical School/Boston Children’s Hospital, Boston, MA • Indiana University School of Medicine, Indianapolis, IN • Medical College of Wisconsin, Milwaukee, WI • Stanford University School of Medicine, Stanford, CA • University of Colorado School of Medicine, Denver, CO • University of North Carolina School of Medicine, Chapel Hill, NC • University of Virginia School of Medicine, Charlottesville, VA • Yale University School of Medicine, New Haven, CT	

## PARTICIPANTS AND METHODS

### Participants

We surveyed all graduates of our one-year faculty development program at the eight schools and received responses from 68 of 92 faculty members (74% response rate). We designed the program to enhance their humanistic teaching and role modeling.^27^^–^29 Site leaders at each school (who are also authors of this paper) selected the participating faculty from a pool of applicants whom they judged to be promising clinical teachers and practitioners.^27^^–^29 Program participants attended twice-monthly experiential and reflective learning sessions for one year.^27^^–^29 All consented to the qualitative study, which was exempted and/or approved by the Institutional Review Board at each institution.

The 68 respondents included 40 (59%) women, 46 (69%) people under the age of 45, and 58 (85%) junior faculty members (instructors or assistant professors). About half of the respondents were primary care internists, pediatricians, or family physicians with the remainder being clinical specialists. The response rate was 11/16 from school #1, 10/10 from #2, 15/17 from #3, 10/10 from #4, 10/12 from #5, 2/17 from #6, 3/3 from #7, and 7/7 from #8. The low response rate from school #6 likely reflects the fact that reminders were not sent to that school’s participants, and the low total number for school #7 reflects a leave of absence by the site leader.

### Data Collection

Program participants at the eight schools were provided a working definition of medical humanism taken from the Arnold P. Gold Foundation^2^ on which to base their answers to our survey. Medical humanism was “characterized by respectful and compassionate relationships among physicians, their patients, and other members of the healthcare team that flourishes within a humanistic culture.” Humanistic healthcare professionals were described as those who “demonstrate integrity, excellence, compassion, altruism, respect, empathy, and service.” Program participants were asked to provide written reflective responses, preferably a paragraph in length, to two prompts: (a) What institutional or specific organizational unit-related factors promote humanism for you and others? and (b) What institutional or specific organizational unit-related factors inhibit or pose barriers, to humanism for you and others? Word counts of the reflective responses averaged 69.6 words (95% confidence 62.6–76.6 words), median 66.0 words, range 3–189 words. Rapid scan of responses by two investigators (WTB and RMF) revealed insightful data worthy of analysis.

### Data Analysis

We employed the constant comparative method^32^^–^35 to analyze the 68 program participants’ responses. Four investigators (WTB, MAG, LGO, EAR) met on six conference calls to analyze responses. Each respondent’s reflection was read aloud on the call, after which the group discussed and reached consensus on the major theme(s) of that reflection. The investigators kept track of themes, and iteratively revised, combined, and/or refined them by consensus as they compared additional sets of writings to those previously analyzed. Illustrative quotes were also identified and grouped under each identified theme.

Investigators agreed that they were coding the themes consistently after comparing their interpretations by the third conference call. By the sixth call, all participant responses had been analyzed and no additional themes were identified. The investigators concluded that they had reached thematic saturation and consensus agreement on the themes. Another author (JPH) independently reviewed and agreed with the choices of themes and related quotes. Final themes are listed in the “RESULTS” section.

## RESULTS

### Motivating Factors for Humanism

Organizational culture was the overarching theme that unified the motivating factors within institutions. Four additional themes were related to, or influenced by, organizational culture: leadership supportive of medical humanism; the responsibility to role model humanism; organized activities designed to promote humanism; and practice structures that facilitated humanism. We noted that many statements pertaining to motivating factors described individual acts and relationships, or participation by small groups of study physicians in activities designed to shore up humanism:

*An organizational culture of humanism:* Respondents described humanistic culture as being maintained by caring relationships that reflected the study physicians’ values. Relationships were about how colleagues treated one another and how they were treated by administration. For example, study physicians described having “*colleagues who are interested in discussing humanism in medicine as a way of supporting each other*,” and “*colleagues [with whom] I can be vulnerable*”; another described a “*professional culture which supports respect, collaboration, and compassion among professionals working together. This culture then hopefully “trickles down” to the doctor patient relationship.*”

Respondents described a level of trust in relationships that encompassed mutual compassion and acceptance of vulnerability. The high level of mutual support between individuals within this culture was illustrated poignantly by the practice described at one institution of calling “*… a ‘code lavender’ that we can call where the chaplain comes to deliver a message, a cup of tea, or just some peace for a particularly challenging day. We debrief every death at the time of the event with all staff involved.*” The sum of these individual relationships characterized by humanistic values 2^,^^12^ contributed to the culture*.* Wrote one participant, “*I am fortunate that humanism and professionalism are core values in my department.*”

*Leadership supportive of humanism:* An illustrative quote described a division chief who exemplified the qualities that study participants associated with good leadership:

“*Humanism is promoted within my division by our division chief. She treats all faculty fairly and with respect. I believe that she sets the tone for our entire division. Because of this, we are better able to work as a team within my division—we support each other through difficult times and cover for each other’s patients whenever necessary. We are given a reasonable amount of time to see patients and a reasonable number of clinic sessions per week. It is my goal as a clinical chief to maintain this degree of supportive environment for all staff who work in our clinic.*”

*Responsibility to role model humanism:* Faculty members reflected that they were always role modeling humanistic qualities for learners. One participant wrote, “*… words and actions, be they large or small, are often being viewed under a microscope by learners. [We have] many young and impressionable learners and it is [our] responsibility to teach them how to provide compassionate care in even the most difficult of situations.*”

Another participant described characteristics of a good humanistic role model: “*The best physicians were those that not only had extensive knowledge and experience, but also those who were humble, listened to everyone’s input, and were comfortable seeking others advice when needed. Role modeling such behavior is important.*”

Yet another reflected that good role modeling could create a ripple effect throughout the organization: “*As a leader, when I show humanism to my faculty, I realize it makes them more likely to show the same to their patients, learners and colleagues … When those to whom I report show humanism to me, this helps me feel that my values are shared with them.*”

*Organized activities designed to promote humanism:* Study physicians believed that some humanistic educational programs helped shape the organizational culture because they, “*allowed for deeper discussions with colleagues*,” and “*allowed the formation of a community of professionals that would reinforce the importance of treating others with respect.*” Examples included a residency curriculum on humanism, programs for underserved populations, reflective writing exercises, regularly scheduled interdisciplinary forums (e.g., Schwartz Rounds), medical student electives fostering compassion and self-knowledge (e.g., The Healer’s Art, Reflection Rounds), and our humanistic faculty development program.^27^^,^^36^^–^40

*Practice structures that facilitated humanism:* A workflow structure that allowed adequate time to build relationships with patients was judged essential. A participant opined, “*Patient workload that is reasonable and conductive to spending the time you would like with each patient rather than always being in a rush.*”

Policies creating structures that facilitated humanistic practice also provided protected time for teaching, adequate staffing to assist physicians with their work, and adequate support available from social workers, case managers, behavioral health professionals, and professional interpreters*.*

Elements of the physical environment that facilitated humanistic culture included co-location of practicing faculty members near their medical assistants and social workers; having a clean and quiet team room; ample chairs; and, in one instance, art work and music.

### Barriers to Humanism

An inhibiting organizational culture disrupted humanistic practice. Additional themes contributing to an inhibitive culture included the following: unsupportive leadership; inadequate time with patients; bureaucratic pressures; and non-facilitative practice structures. Some barriers resulted from individual actions, but often barriers reflected system-related factors, business practices, and bureaucratic requirements.

*Inhibiting organizational culture:* An inhibiting organizational culture was described by two participants as occurring when, “*colleagues treat patients or patient’s concerns with disrespect or interact…in an arrogant or dismissive fashion*,” or where “*there is not much of a culture of sharing personal stories and recognizing the humanity of colleagues.*”

Sustaining humanistic culture required unified efforts from faculty members, leaders, and staff that often did not occur. One participant put it this way, “*[It is…difficult for me to gain trust when some members of my team are uncaring.*” Behavior that overtly impeded humanism was sometimes described in terms of, “*disruptive personalities that bully colleagues and students.*”

Several respondents pointed out that maintaining a humanistic culture was an ongoing process that should “*not be taken for granted*” and required unremitting efforts. Leaders should, “*recognize that we need to be nourished in mind and spirit.*”

*Unsupportive leadership:* For leadership to be out of touch with the day-to-day struggles of physicians, or to make productivity the overriding focus, impeded humanistic culture. One participant summed it up this way: “*I think leadership…do[sic] not always listen to what a provider requires to be humanistic when it may compete with another goal such as efficiency or increasing the number of patients that are seen*,” and when the leadership is “*prioritizing RVUs over patient centered care*.” Thus, excessive focus on productivity was of special concern when done at the expense of physicians’ ability to deliver humanistic care. As one writer stated, “*When the focus is on productivity, when the institution provides no tangible value for taking the time or initiative to be humanistic, a barrier is slowly, but inevitably built*.”

Participants identified unrealistic expectations and inadequate support personnel as creating an unsupportive environment. For example, one participant stated, “*At times, the administration of the hospital doesn’t have a thorough understanding of the struggles that are going on in various care settings of the institution. Physicians can be asked to adopt too many initiatives at once leading to frustration and negativity. In addition, some treatment areas receive less support/resources in terms of nursing or administrative personnel. Being understaffed can create an environment of stress and negativity—leading to a less humanistic culture within the institution.*”

Unsupportive or out of touch leadership was resented. One study participant wrote that it was, “*… about not being respected—not understanding or realizing the stresses I am going through.*”

*Time and bureaucratic pressures:* Many respondents mentioned “*time*” as a barrier*.* Workflow that provided insufficient time with patients and pressure to generate RVUs often drove dissatisfaction as reflected in the following quotes:

“*Time and bureaucratic pressures are important factors. Adequate time needs to be given for patient contact, record-keeping, ancillary contact, consultation, self-care (meals, breaks) and breathing space between times of patient-contact*.”

“*The whole approach to reimbursing physicians using RVUs, a system that is biased toward doing procedures and not spending time with patients…I think this is the biggest barrier right now to treating people humanistically*.”

*Non-facilitative practice structures:* The study physicians lumped together bureaucratic pressures, out of touch leadership and poorly structured practice workflow as producers of stress, frustration and dissatisfaction. One writer commented, “*Too many responsibilities, no admin support, disconnected units, parts that don’t communicate with others*.”

Physical space was sometimes viewed as non-facilitative. A participant identified, “*small hospital rooms, no family rooms, team rooms that are away from the patient care areas.*”

The EMR was often singled out as a barrier. “*Our EMR does more to retard humanism than any recent change in practice, from the mere reality of looking at a screen rather than the patient in the room, or knowing you’ll spend twice or 3 times the time documenting as you could possibly spend caring for or ‘hearing’ the patient.*”

## DISCUSSION

Our study’s results highlight the effects of organizational culture on humanistic medical practice. We know that organizational culture influences patient safety, quality of care, medical errors, patients’ and families’ experiences of care, physician satisfaction, and burnout.^21^^,^^24^^,^^41^^–^43 Our qualitative study adds real life meaning to these reports by linking them to the lived experiences of its study physicians. Study physicians named supportive colleagues, influential role models, engaged leaders, and adequate time to build relationships with their patients as factors that sustained their medical humanism. Because it highlighted discordances between individual physicians’ goals to build therapeutic relationships with their patients, and prevailing system barriers and bureaucratic requirements, the study suggests the need for transforming organizational culture to make it more sustaining of humanistic practice. Strategies to reinforce the humanistic and relational aspects of care and their alignment with an organization’s values^12^^,^^23^^,^^27^^,^^38^^,^^39^^,^^41^^,^^42^^,^^44^^–^46 are needed if an organizational culture of humanism is to flourish.^43^^,^^47^^–^49

**Figure 1:**
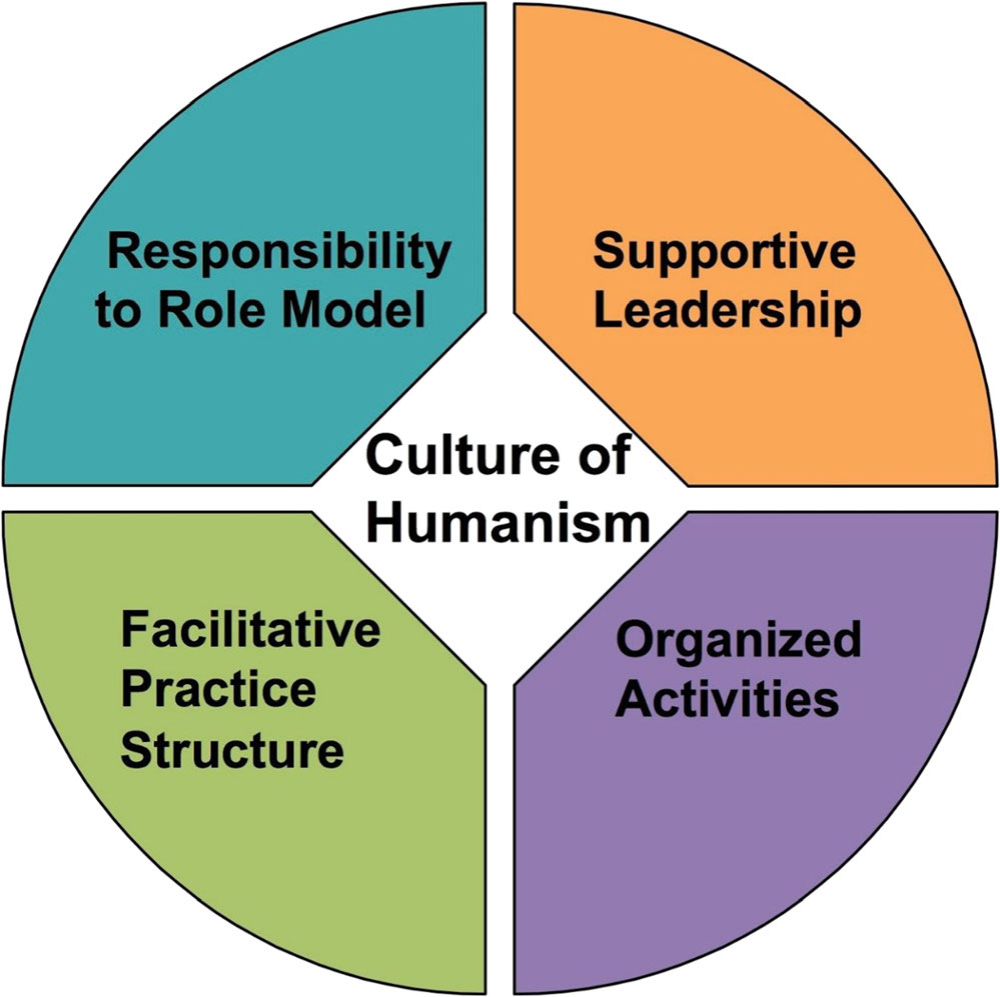
Organizational-level factors identified as promoters of humanistic practice.

Although often assumed to be important in shaping performance, organizational culture can be difficult to define.^48^ One widely shared viewpoint defines organizational culture as a pattern of shared assumptions and correct ways to perceive, think, and feel in relation to problems.^47^^,^^48^ Our study physicians added that culture reflected the qualities of their relationships with colleagues and patients. Relationships operated through people within their organizations to shape and influence the other factors, such as workflow designs, and the alignments of leaders’ and physicians’ goals and values.^49^ Figure 1, reflecting the themes expressed by our study physicians, provides a graphic representation of positive components in organizations that were identified as contributing to an organizational culture of humanism. Our study suggested ways to achieve this humanistic vision, but it also implied blind spots and inadequacies that must be overcome by physicians and leaders who wish to change their organizations for the better.

### It Is Time for Organizational Change

The study uncovered high levels of physician dissatisfaction with a number of health care systems. We attribute the dissatisfaction to incongruence between the study physicians’ core humanistic values and the current business climate and bureaucratic requirements to which they are subjected.^45^^,^^46^ The study physicians described a clash of disparate cultures.^50^^–^53 These included the tone and attitude of some administrators, the metrics used to judge physicians’ performance and, most of all, the pressures of time and numbers of patients seen. Resultant stress, frustrations, dissatisfaction, and burnout call for major change.^21^^,^^30^^,^^43^^–^45^,^^53^

### Leadership is Key

Supportive leaders should effectively model change-behavior and/or articulate a clear vision of necessary changes.^47^ Study participants described good leaders as “walking the talk,” by treating people fairly and respectfully, promoting and valuing humanism in patient care, and advocating for adequate time with patients, better use of teamwork, and sufficient support staff. Good leaders protected faculty members from unrealistic or intrusive requirements and productivity pressures. Not all leaders were described this way; some were “disengaged” from the physicians’ struggles.

We also think that more is required of today’s leaders than to bolster equanimity within an apparently unsatisfactory status quo. Experts on organizational behavior identify leadership as a crucial element to create, sustain and, at times, change organizational culture^24^^,^^47^ If elements of a given culture become dysfunctional, “… leadership has to surmount culture and speed up the normal evolution with forced cultural change programs.”^47^

### It Is Not All Up to the Leaders

Study participants identified their individual role modeling as an effective way to foster humanistic practices.^54^^–^56 They participated in educational programs with like-minded colleagues designed to enhance humanism, strengthen resilience, and promote well-being.^6^^,^^27^^,^^36^^–^40 Less formal activities included family conferences, multidisciplinary case conferences, check-ins, and opportunities for story-telling. As an additional example, Indiana University School of Medicine employed multiple small steps that called attention to respectful, collaborative relationships as the foundation of humanistic interactions.^57^^,^^58^ These are downstream activities that may assist physicians in dealing with the stress without altering the practice.

This leads to the observation that physicians, like those we studied, were acting individually to be humanistic but were not joining collectively with their leaders to create system changes and an organizational culture that delivers excellent, safe, efficient care and preserves humanistic values.^46^ Physicians may be overlooking the basic principle of working together to create organizational change.

### Recommendations

Complex organizational cultures are co-created by administrative and managerial leaders, physicians, and other healthcare professionals, staff, and stakeholders. We will focus here on organizational change strategies as a prelude to changing practice structures. The first step in positive change is for physicians, leaders, and all stakeholders to reach a consensus on the organization’s mission, strategy, and goals.^47^ The challenge begins with each organization’s leaders. Our study physicians described some leaders as good role models and supportive advocates, and others less so, but strikingly absent was language describing the leaders as change agents, articulating a clear vision for how to reshape and improve the organization.^12^^–^15^,^^41^^,^^42^^,^^59^ Our observations of study physicians’ responses to our survey lead to a related question. Although the study physicians identified individual relationships as well as programs and practice structures that promoted humanism, they never mentioned sharing a common vision. We suggest that a shared vision of the practice is a necessary first step to bring the elements of Figure 1 into being. Faculty members, leaders, other healthcare professionals, and staff may then collectively shape their organization and its culture to achieve their vision, which ought to encompass a community of practice congruent with professional values and maximally beneficial to patients. We judge our study physicians to be deeply engaged with their individual patients and humanistic relationships with learners and colleagues, yet potentially missing a strategic organizational focus. This applies equally to leaders. Both groups need to build their relationships and work together to effectively produce desirable structural changes that create and preserve humanism in organizations. In the Appendix online, Table 1, we describe attributes and best practices that might characterize a humanistic organization. Our recommendations are based on this study’s results and our collective experience as medical educators. Specific details for re-structuring medical practices to enhance physician satisfaction are being tried and studied.^60^^,^^61^ We thought it premature to propose concrete changes in the workflow, team functioning, and other practice structures in Table 1 in the Appendix, online. They are important subjects for continuing research.

### Limitations

Because the study participants were volunteers who completed a year-long program in humanism, they may not represent all faculty members within or outside of teaching institutions. Health care organization was not a topic in our faculty development course on humanistic role modeling and teaching. We doubt that our course primed our study physicians’ views of organizational motivators and barriers to medical humanism. This was a qualitative study subject to selection bias. However, as promising and respected teachers with an interest in humanism, we expected the study physicians to have insights and to be humanistic leaders who could suggest the way forward for their organizations. Although some of our conclusions may be less generalizable to practices and physicians outside of teaching environments, we believe that our findings, including, for example, the importance of working together to establish a culture of humanism, explicitly role modeling humanistic practice, and providing supportive practice structures, should be applicable to a variety of patient care settings. Our qualitative approach uncovers root causes and processes, a necessary step for improving practices. To confirm our results would require a larger study using quantitative methods.

## CONCLUSIONS

Efforts to counteract physician dissatisfaction, burnout, and other negative consequences of recent changes in medical practice have largely focused on supporting and cultivating humanistic attributes of individual physicians.^62^ Our study findings suggest that addressing organizational factors is at least equally important. Without positive organizational changes, actions focusing only on individuals are unlikely to achieve physician satisfaction, resilience, compassion, and well-being along with optimal patient care.

## Electronic supplementary material

ESM 1 (DOCX 17.4 kb)
