# Supplementary material for: Healthcare at the Crossroads: The Need to Shape an Organizational Culture of Humanistic Teaching and Practice
Source: J Gen Intern Med. 2018 May 8;33(7):1092–9. doi: 10.1007/s11606-018-4470-2 (PMC6025655; doi:10.1007/s11606-018-4470-2)
Supplement: Supplementary file 1 — (DOCX 17.4 kb) [file 11606_2018_4470_MOESM1_ESM.docx]

| **Concepts/Themes** | **Characteristics** |
| --- | --- |
| ***Attention to values*** | - The professional culture supports respect, collaboration, and compassion among professionals working together. - Excellence expected and role modeled by senior leadership. - Institution cares about those it serves. - Institution provides generous charity care. - Humanism and professionalism are core values in the department. - Medical school faculty and leaders have humanistic values. - Relationships and connection with patients is valued over RVUs and volume of care. |
| ***Relationships are seen as important*** | ***With patients and families***   - Relationships characterized by reciprocal influence - Family members have a voice and family engagement is encouraged.   - Families attend group meetings quarterly; inform physicians about their priorities and perceptions of care   - Family advisory council members attend clinic council meetings - Patient feedback is collected as to whether patients’ needs are being met.   ***With colleagues / team members***   - Individual relationships with other professionals and a shared sense of pride in the work we do are important. - Having great colleagues with whom one can be vulnerable - Colleagues are interested in discussing humanism in medicine as a way of supporting each other. - Opportunities to talk and share our stories - Shared expectation of how we treat and interact with patients that encourages a humanistic approach - Learning and support from social workers, nurses, chaplains, case managers - Adding more progressive-thinking individuals and diversity in higher ranked positions - Significant strides in interpersonal skills and how we treat each other on campus |
| ***Good leadership*** | - Leaders (department chairs, division chiefs) promote humanism and set the tone. - Leaders work with all stakeholders to create a shared vision of a humanistic practice that delivers excellent, cost-effective, safe patient care. - Role models in departmental and school of medicine leadership value humanism and thoughtful teaching. - From the highest level of leadership there is a focus and recognition of the importance of this aspect of patient care and education. - Good communication creates understanding between hospital leadership and staff. - Hospital administration has a thorough understanding of the needs and of the struggles occurring in various care settings of the institution. - Leaders both “talk the talk and walk the walk.” - Leadership and all stakeholders work together to implement system changes that promote safe, cost-effective, excellent, humanistic care. |
| ***Commitment to humanistic teaching and learning environments*** | - Humanistic faculty role models are recognized and rewarded. - Learning community that helps model ideal professionalism in teaching and being humanistic - Teaching conferences and direct supervision of residents and students provide opportunities to demonstrate team effort and to promote humanism. - Departmental awards/recognition for being good teachers/role models - Ongoing education is a priority. |
| ***Facilitative practice structures*** | ***Time with patients***   - Physicians are allowed adequate time to build relationships with their patients and have long conversations if needed. - Patient workload is reasonable and conductive to spending the time you would like with each patient rather than always being in a rush. - Reasonable clinical volume and adequate time to see patients - Sufficient number of attending physicians to promote taking the time to address patient and family questions - Decreased pressure for productivity, allowing a focus on excellence.   ***Professional Autonomy***   - Physicians have flexibility and say over visit times with patients.   ***Time to teach***   - Sufficient number of attending physicians to promote taking the time to teach - Protected time for teaching is supported as a core part of one’s work –“not a hassle that takes me away from revenue generating processing.” - A patient load that is less than what would exist in private practice so that one can spend time teaching, learning, and demonstrating skills - Protected time each week to devote to teaching   ***Workload***   - Adequate time is given for patient contact, record-keeping, ancillary contact, consultation, self-care (meals, breaks) and breathing space between times of patient-contact. - Sufficient non-clinical time to allow faculty development and growth specifically devoted to humanism - Sufficient night and daytime attending coverage allows for less fatigue, as there is a fresh person for each shift, and encourages self-care. - Attention to innovative ways to diminish time with EMR and other record keeping activities that take time from direct patient care   ***Sufficient Support Staff***   - Good administrative support and efficiency - Support staff carry out appropriate tasks allowing physicians to spend more time with patients.   ***Support for teamwork / adequate resources***   - A team room or space “that brings us together and we can function more as a team” - Presence of social workers on rounding teams - Social workers and case managers help advocate for patients. - Access to resources for patients, including social work, behavioral health, and support groups - When setting production benchmarks, use accounting systems that do not penalize providers for caring for patients who are unable to pay for their care. |
| ***Support for good work-life balance and well being*** | - Support is given for childcare, time away for personal or family illness, and subsidies are provided for education. - Sufficient non-clinical time allows faculty development and growth specifically devoted to humanism. - Recognition “that we need to be nourished in mind and spirit” |
| ***Organized Programs and Activities*** | (See text and references) |

**Appendix (Online), Table 1.** Suggestions for Achieving an Organizational Culture of Humanism

From: Rider EA, Gilligan MC, Osterberg LG, Litzelman DK, Plews-Ogan M, Weil AB, Dunne DW, Hafler JP, May NB, Derse AR, Frankel RM, Branch WT Jr. **Healthcare at the Crossroads: The Need to Shape an Organizational Culture of Humanistic Teaching and Practice.** J Gen Intern Med. 2018.
